# Intramolecular Hydrogen Bonds in Conformers of Quinine and Quinidine: An HF, MP2 and DFT Study

**DOI:** 10.3390/molecules22020245

**Published:** 2017-02-07

**Authors:** Mireille K. Bilonda, Liliana Mammino

**Affiliations:** Department of Chemistry, University of Venda, Thohoyandou 0950, South Africa; mireillebilonda@yahoo.fr

**Keywords:** alkaloids, antimalarials, conformers’ stabilising factors, intramolecular hydrogen bonding, quinidine, quinine

## Abstract

Quinine is an alkaloid with powerful antimalarial activity, isolated from the bark of Peru’s cinchona trees. Quinidine is an erythro diastereoisomer of quinine also exhibiting antimalarial activity. Conformational studies performed so far had never identified conformers with intramolecular hydrogen bonds (IHB). The current study shows the possibility of conformers with an IHB between the quinuclidine and quinoline moieties of these molecules. The study was performed at different levels of theory: Hartree Fock (HF) with the 6-31G(d,p) basis set, Density Functional Theory (DFT) with the B3LYP functional and the 6-31+G(d,p) basis set and Møller–Plesset Perturbation Theory (MP2) with the 6-31+G(d,p) basis set, to confirm the results. The results suggest that the stabilising effect of this IHB is weaker or comparable with respect to the stabilising effect of the preferred mutual orientation of the two moieties. Although the IHB-containing conformers may not be the lowest energy ones, their relative energy is sufficiently low for them to be included among the possible ones responsible for the compounds’ antimalarial activity.

## 1. Introduction

**Figure 1:**
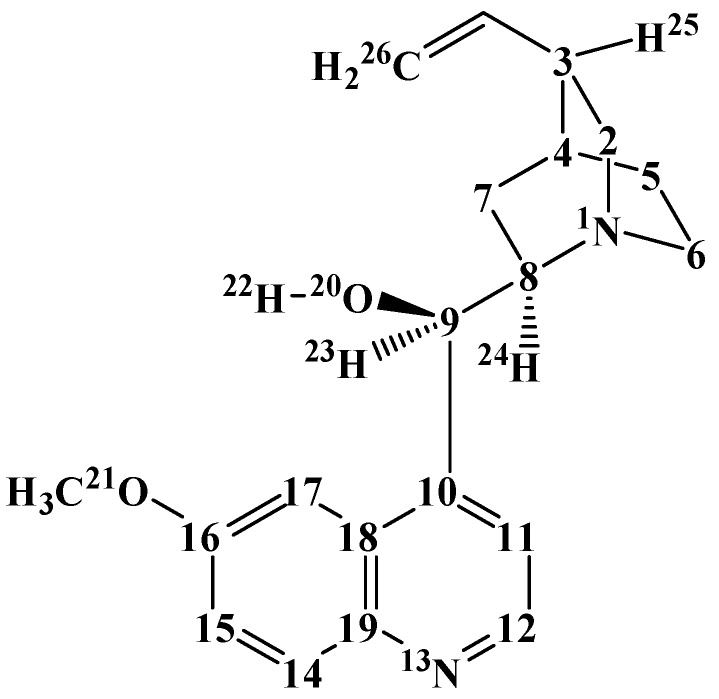
Structure of the quinine molecule and atom numbering utilized in this work. The C atoms are represented only by the numbers denoting their positions. The H atoms attached to C atoms in the rings are not shown to better highlight the molecular structure. For the same reason, the H atoms attached to terminal methyls are not numbered individually (they do not need to be mentioned individually in the analysis of results).

Quinine (C_20_H_24_N_2_H_2_, Figure 1) and quinidine are alkaloids isolated from the bark of cinchona trees found in Peru. Like all cinchona alkaloids, their molecules contain two moieties—a quinuclidine moiety and a quinoline moiety. Quinidine is an erythro diastereoisomer of quinine; the absolute configuration of quinine is 1*S*,3*R*,4*S*,8*S*,9*R* and that of quinidine is 1*S*,3*R*,4*S*,8*R*,9*S* [1]. Both compounds are active against malaria. Quinine has been the most powerful known antimalarial for a long time and is still a resource drug for the treatment of malaria [2]; its use is currently reserved for particularly severe cases in order to prevent the development of resistance by the *plasmodium* parasites [3]. Quinidine is also used against cardiac arrhythmias [4]. Because of its pharmaceutical importance, quinine has been the major object in the studies focusing on cinchona alkaloids.

Figure 1 shows the atom numbering utilised in this work. The most relevant rotatable bonds in cinchona alkaloids are those linking the quinuclidine and quinoline moieties, i.e., C8–C9 and C9–C10, corresponding to the C11–C10–C9–C8, C10–C9–C8–N1 and H22–O20–C9–C8 torsion angles. Their rotations determine the mutual orientations of the two moieties, thus also determining the possibility that the N atom and the OH group come close enough to form an intramolecular hydrogen bond (IHB). The orientations of the hydroxyl group and of the methoxy group may also influence conformers’ energy [5,6].

Since early experimental [7,8,9,10,11] and computational studies, the mutual orientation of the two moieties has been identified as a major factor influencing conformers’ stability. A molecular mechanics (MM2P) study identified three main conformations, corresponding to three different angles for the rotations around the C8–C9 and C9–C10 bonds [8,11]. Another MM2 study [11,12] identified the same three conformers; the lowest energy one (which was denoted as Q1) was found to correspond to the crystal structure of quinine and to make intermolecular H-bonds with surrounding molecules [11,13]; the authors presumed that Q1 is the active conformer and it interacts with the receptor site through intermolecular H-bonds. Studies using MM and the AM1 and PM3 semi-empirical methods [11] identified six minima on a multidimensional Potential Energy Surface (PES), with the three lowest ones corresponding to those of [7] and having energy gaps less than 2 kcal/mol. From the results of ab initio calculations with the 3-21G basis set and the consideration of electron density distribution and other molecular properties [14], Karle and Bhattacharjee suggested that, for a cinchona alkaloid to have antimalarial activity, it should “contain a hydroxyl group of sufficient acidity and quinoline and aliphatic nitrogen atoms that are not too basic” and the orientations of the OH group and the part around the N in the quinuclidine moiety play a role, “most likely by controlling the ability of these groups to form effective intermolecular hydrogen bonds”; they also indicated that a potent antimalarial cinchona alkaloid has a dipole moment of 2.2 D or less, an electric field pointing from the aliphatic nitrogen atom towards the aromatic ring, an acidity equivalent to at least 34.7 kcal/mol of positive potential by the hydroxyl proton, a basicity of negative potential by the aliphatic nitrogen atom of no more negative than −64.4 kcal/mol, a proton affinity of the aliphatic quinuclidine nitrogen atom of 200 ± 5 kcal/mol, and a comparatively moderate electron affinity. Another PM3 study [1] classified the conformers in terms of their C11–C10–C9–C8, C10–C9–C8–N1 and H22–O20–C9–C8 torsion angles.

The results of a B3LYP/6-31+G(d,p) study of quinine and quinidine [15] were also analysed in terms of the C11–C10–C9–C8, C10–C9–C8–N1 and N1–C8–C9–O20 dihedral angles. The values of the C11–C10–C9–C8 and C10–C9–C8–N1 were utilised to classify geometries as *closed* or *open* (as initially introduced in [1]), where *close* corresponds to ≈50° and *open* corresponds to ≈150° or ≈290°. The value of the C8–C9–O20–H22 dihedral angle was utilised for a classification denoted as α, β and γ (as introduced in [15]), respectively corresponding to +60°, −60° and 180°. The orientation of the methoxy group of the quinoline ring relative to the C9–C10 bond was ascribed the symbols *cis* if the methyl of that group is oriented towards the other ring and *trans* if it is oriented away from the other ring. In this way, quinine has the conformers *cis*-γ-open, *cis*-α-open, *cis*-γ-closed and *trans*-γ-open with *cis*-γ-open being the most stable (72% population). No IHB was identified in the conformers of quinine. For quinidine, the most stable conformer (78% population) corresponded with a *cis* open geometry with a −153° C10–C9–C8–N1 dihedral angle. Because of their importance for the classification of conformers, the values of the C11–C10–C9–C8, C10–C9–C8–N1 and H22–O20–C9–C8 torsion angles from these studies are shown in Table 1.

Figure 1 in [15] showed that all the conformers considered there had the OH oriented towards the quinoline moiety. This motivated the current study, as we assumed that the opposite orientation of the OH might enable the formation of an IHB with the N atom of the quinuclidine moiety.

## 2. Computational Details

Calculations in vacuo were performed at the following levels: Hartree Fock (HF) with the 6-31G(d,p) basis set, Density Functional Theory (DFT) with the Becke, 3-parameter, Lee-Yang-Parr hybrid functional (B3LYP, [16,17] and the 6-31+G(d,p) basis set and Møller–Plesset Perturbation Theory (MP2) with the 6-31+G(d,p) basis set, all with fully relaxed geometry. The HF method is a common and less expensive quantum method that can yield accurate information regarding conformational analysis. HF can handle intramolecular hydrogen bonding, as shown in previous studies of other molecules [18,19,20,21]. DFT is an alternative method to wavefunction approaches, which is often used in a conformational search because it takes into account part of the correlation effects at a comparatively low cost. Among the numerous functionals available for the DFT framework, B3LYP is the most widely employed. It can provide better quality results in combination with basis sets containing diffuse functions. The MP2 method [22] takes into account both the electron correlation and the dispersion energy, and is an efficient level of theory for the evaluation of energy differences between minima. For a better description of the dispersion energy, the basis set should contain polarized and diffuse functions that account for molecular polarizability and the tendency of electrons to diffuse from a high electrons density region to a low electron density region [23,24]. Apparently, 6-31+G(d,p) is the smallest basis set that can yield reliable information on hydrogen bonding with higher levels of theory such as MP2 and DFT, and it is convenient to use because larger basis sets tend to increase the computational time and can lead to convergence problems of the total electronic wavefunction of the molecular system.

Vibrational frequencies (harmonic approximation) were calculated in vacuo at the HF and DFT levels, and scaled by the factors recommended for these two methods [25,26]. They were not calculated with MP2 because the HF frequencies (after scaling) are better than the considerably more expensive MP2 ones [23].

Calculations in solution utilised the Polarizable Continuum Model (PCM, [27,28,29,30,31,32]). In this model, the solvent is modelled by a continuous isotropic dielectric. The solute molecule inserts itself into the solvent, generating a cavity within which it gets embedded. The cavity thus corresponds to the space occupied by the solute molecule within the continuum solvent. The geometry of the cavity follows the geometry of the solute molecule, considering its solvent accessible surface. The default settings of Gaussian03 [33] were utilised, namely: Integral Equation Formalism model (IEF, [29,30,31,32,33]) and GEPOL (Generate-polygon procedure in molecular surface tessellation) model for building the cavity around the solute molecule [34,35,36], with simple United Atom Topological Model (UAO) for the atomic radii and 0.200 Å2 for the average area of the tesserae into which the cavity surface is subdivided. The SCFVAC input option was selected to obtain more thermodynamic data through the consideration of preliminary gas-phase energies.

Calculations in solution were performed as single point (SP) calculations on the in-vacuo optimised geometries, with the same levels of theory utilised in vacuo. It was opted to use SP calculations because the size of the molecule makes re-optimisation in solution computationally expensive. Although SP calculations cannot provide information on the changes in the geometry of the molecule caused by the solvent, they can provide reasonable information on the energetics, such as the conformers’ relative energies in solution and the energy aspects of the solution process (the free energy of solvation, ΔG_solv_, and its components). In this work, the most important information concerns the relative energy of the conformers with the IHB in solution and, therefore, SP calculations are sufficiently informative. Two solvents were considered—chloroform and water—to mimic the most important media within living organisms: the non-polar medium (mostly, lipid phase in the body) and water, which creates a polar medium and is the major component of living organisms. Chloroform is an apolar aprotic solvent with low relative permittivity (ε_r_ = 4.90) and low dipole moment (µ = 1.04 D [37]). Water is a protic solvent with high relative permittivity (ε_r_ = 78.39) and a sizeable dipole moment (µ = 1.83 D [37]).

Calculations were performed using GAUSSIAN 03 (Gaussian Inc., Pittsburgh, PA, USA), Revision D 01 [33].

All the energy values reported are in kcal/mol and all the distances are in Å. Acronyms are utilized for the calculation methods and for the media, for conciseness sake, on reporting values: HF for HF/6-31G(d,p), DFT for DFT/B3LYP/6-31 + G(d,p), MP2 for MP2/6-31 + G(d,p), ‘vac’ for vacuum, ‘chlrf’ for chloroform, and ‘aq’ for water.

## 3. Results

### 3.1. Results in Vacuo

Besides the conformers expected to have an IHB, the conformer corresponding to *cis*-γ-open(3) in [15] (the lowest energy conformer of all the previous studies, called Q1 in the earlier studies) was also calculated, as a reference to compare the energy of the conformers with the IHB. Both the *cis* and *trans* options, as defined in [15], were calculated for each type of conformer to verify possible influences from the orientation of the methoxy group.

**Figure 2:**
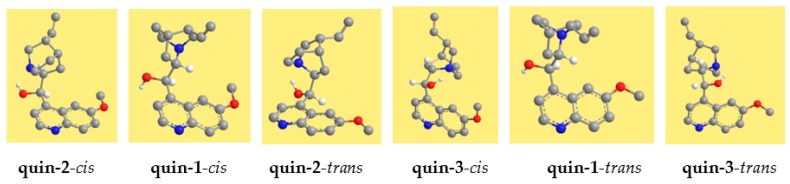
Conformers of quinine calculated in this work. Density Functional Theory (DFT/6-31+G(d,p)) results. The conformers are shown in order of increasing relative energy.

**Figure 3:**
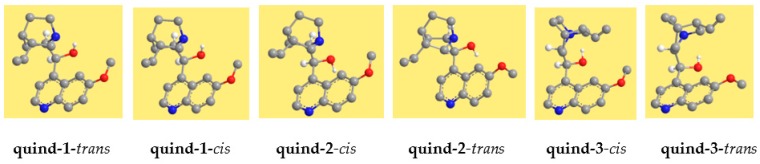
Conformers of quinidine calculated in this work. DFT/6-31+G(d,p) results. The *cis* and *trans* forms of the same pair are shown nearby to facilitate their comparisons.

The resulting conformers are shown in Figure 2 for quinine and in Figure 3 for quinidine, and their relative energies are reported in Table 2. The conformers are denoted with acronyms starting with ‘quin’ for quinine and ‘quind’ for quinidine. This is followed by a number, increasing according to the relative energy in the DFT results. The relative energy sequence for quinine is considerably different in the results of the different methods utilised. However, the spectroscopic experimental information reported in [15] shows that the lowest energy conformer does not have an IHB. Therefore, it is opted here to choose a relative energy sequence consistent with this, in assigning numbers to conformers. DFT results are chosen with preference to HF results because DFT takes into account part of the electronic correlation. Furthermore, they are closer to MP2 results, which take into account both electron correlation and dispersion energy. The conformers with the IHB (quin-2-c, quin-2-t, quin-3-c and quin 3-t for quinine and quind-1-t, quind-1-c, quind-3-c and quind-3-t for quinidine) have relative energy only marginally different from those without the IHB in the MP2 results, whereas the difference is not negligible in the DFT results, but also not so large as to exclude them from being potentially responsible for the biological activity. This consistency in the DFT and MP2 results in terms of yielding a low enough relative energy for the conformers with the IHB to include them as potentially responsible for the biological activity can be considered as a clear indication that such conformers need to be included.

In the acronym denoting the conformers, the number is followed by ‘c’ for *cis* or ‘t’ for *trans*. For quinine, *cis*-γ-open(3) corresponds to Q1 in [15]; for quinidine, quind-2-t corresponds with the conformer with the lowest relative free energy in [15]. For quinine, *cis* conformers have lower energy than corresponding *trans* conformers, whereas for quinidine, *trans* conformers have lower energy. For both molecules, the difference between corresponding *cis* and *trans* is considerable. For quinine, this energy difference (kcal/mol) is ≈2/HF, 1.6–3/MP2 and ≈1/DFT; for quinidine, it is 2–3/HF, 1–5/MP2 and 1–3/DFT.

Table 3 reports the relative free energies (∆G, sums of electronic and thermal free energies) of the calculated conformers in the HF and DFT results in vacuo. For quinine, the conformer with the lowest relative energy (quin-1-c, not having an IHB) also has the lowest ∆G. For quinidine, the quind-1-c conformer (having the IHB) has the lowest ∆G. Comparisons with the ∆G values reported in [15] is not easy because only the lowest energy conformer of [15] was calculated in this work, and, therefore, the comparison can refer only to this conformer. For quinine, the conformer with the lowest ∆G coincides in the current results and in [15]; for quinidine, a different conformer (quin-3-c, not present in [15] and with an H···N distance too long to be considered an IHB but still suggesting a weak interaction between the two atoms) has the lowest ∆G in the current results.

Table 4 reports the relevant torsion angles, i.e., the torsion angles indicating the mutual orientations of the ring systems (C11–C10–C9–C8 and C10–C9–C8–N1) and the orientation of the OH group (H22–O20–C9–C8 and O20–C9–C8–N1). The orientation of the methoxy group does not influence the rest of the geometry significantly, and corresponding pairs of *cis* and *trans* conformers have close values for these torsion angles. Conformers 1 and 2 differ for the C10–C9–C8–N1 torsion angle, and conformers 2 and 3 differ for the C11–C10–C9–C8 torsion angle; both angles are associated with the orientation of the two moieties.

Table 5 reports the parameters of the IHBs. The H···N bond length would suggest a moderate strength for the IHB [38]. However, the directionality, as shown by the NĤO angle, is not optimal, which may contribute to decreasing the strength. The parameters indicate a stronger IHB for quinine than for quinidine, and the directionality is also more favourable for quinine. Comparison of the three methods shows that MP2 optimises for geometries with better IHB parameters.

Frequency calculations confirm that the identified stationary points correspond to true minima. Table 6 reports the vibrational frequencies of the OH bond (harmonic approximation) in the HF and DFT results. With reference to the 3650 cm^−1^ experimental frequency value for the ground state reported in [15], the HF results obtained here are considerably higher, whereas the DFT result obtained here (3766 cm^−1^), although being 116 cm^−1^ higher than the experimental value, is closer to the experimental value than the calculated result reported in [15].

It is interesting to consider the decrease in the vibrational frequency caused by the formation of the IHB (red shift). The red shift is evaluated with reference to the vibration when the OH is not engaged in the IHB. Although the frequency of quin-1-c and quin-1-t, or quind-2-c and quind-2-t, are very close, it was opted to evaluate the red shifts of the *cis* conformers with respect to the frequency of the OH in quin-1-c and quind-2-c, and the red shifts of the *trans* conformers with respect to the frequency in quin-1-t and quind-2-t. The red shifts are reported in Table 7. For quinine, the value of the red shift is 67–98 cm^−1^/HF and about 250 cm^−1^/DFT; for the quind-1-c and quind-1-t conformers of quinidine, it is 63 and 68 cm^−1^/HF and 210 and 248 cm^−1^/DFT, respectively. The quind-3 conformers do not show any red shift with respect to quind-2, and the HF and DFT N···H values are longer than what was normally accepted for an H-bond, although they suggest the presence of a weak interaction between the two atoms (only the MP2 values suggest an IHB).

Table 8 reports the dipole moment of the calculated conformers. The dipole moment of the conformers with the IHB is significantly greater than for that of conformers without the IHB for both quinine and quinidine and in the results of all the methods.

### 3.2. Results in Solution

Table 2 also shows the relative energies in chloroform and water solutions. For quinine, the increasing relative-energy sequence remains the same in vacuo and in solution. For the lower energy conformers of quinine having the IHB, the relative energy is lower in chloroform than in vacuo; in water, it is higher than in chloroform and, for some conformers, it becomes close or slightly higher than in vacuo. For quinidine, the increasing relative-energy sequence changes in solution. The lowest energy conformer is a conformer with the IHB both in vacuo and in chloroform (quind-1-t and quind-1-c respectively), whereas quind-3-c (with a very weak IHB-type interaction) is the lowest energy conformer in water. The relative energy increases in solution with respect to in vacuo for *trans* conformers and decreases for *cis* conformers, up to the point that, differently from in vacuo, *trans* conformers have higher relative energy than the corresponding *cis* conformers in solution. For conformers quind-1-t, quind-1-c and quind-2-c, the relative energy is higher in water than in chloroform; for the other three conformers, it is lower in water than in chloroform.

The dipole moment (Table 8) increases as the medium polarity increases (which is the most common trend). Since calculations in solution were SP calculations, the increase is due only to the polarization of the solute molecule by the solvent (not to a change in the molecular geometry). The dipole moment of the conformers with the IHB remains higher than that of conformers without the IHB in all the media and with all the calculation methods, both for quinine and for quinidine. The *cis* conformers have higher dipole moment than the *trans* conformers in all the media and with all the levels of theory, for both quinine and quinidine.

Table 9 shows the free energy of solvation (ΔG_solv_) of the calculated conformers. The presence or absence of the IHB does not appear to influence ΔG_solv_. The magnitude of ΔG_solv_ is much greater for water than for chloroform. For quinine, ΔG_solv_ is negative for all the conformers and with all the calculation methods. For quinidine, ΔG_solv_ is negative in water for all the conformers; in chloroform, it is negative for most conformers but positive for quind-2-c in the HF and DFT results. For quinine, ΔG_solv_ is smaller for *cis* conformers than for *trans* conformers in all the solvents and with all the calculation methods. For quinidine, ΔG_solv_ is greater for quind-3-c and quind-1-c than for their corresponding *trans* conformers, whereas it is smaller for quind-2-c than for quind-2-t in both solvents and with all the calculation methods.

Table 10 reports the electrostatic component (G_el_) of the free energy of solvation (ΔG_solv_) of the calculated conformers. G_el_ has negative values in both solvents for all the conformers, and its magnitude is considerably greater in water than in chloroform. The non-electrostatic component of ΔG_solv_ can be easily calculated as G_non-el_ = ΔG_solv_ − G_el_.

## 4. Discussion

The most important result of this study is to have shown that quinine and quinidine may form an IHB whose strength is likely between weak and moderate (with the IHB length pointing towards ‘moderate’ and the NĤO angle being closer to the ‘weak’ range). The fact that three different computational methods all show the possibility of the IHB confirms the result. The relative energy values show that the stabilising effect of the IHB may be comparable to the stabilising effect of preferred mutual orientations of the two moieties for the lowest energy conformers in which it is present.

The conformer responsible for the biological activity of a compound is not always the lowest energy one. It may be one with higher relative energy, but still with a sufficient population to be able to exert the activity. Different authors often use different threshold values as the maximum energy value beyond which a conformer is not likely to be involved in the biological activity. A very cautious threshold value is 3.5 kcal/mol; thus, conformers whose relative energy is not ≤3.5 kcal/mol in at least one of the media can be excluded. The results of this study (Table 2) show that both the lower energy conformer with the IHB and the lower energy conformer without the IHB might be involved in the biological activity. If one considers the MP2 and DFT results, only quin-3-t is excluded from being possibly responsible for the biological activity. Thus, the relative energies of the conformers with the IHB (quin-2 and quin-3) in the various media show that these conformers may be involved in the biological activity of quinine. For quinidine, if one considers the DFT results, no conformer is excluded from being one possibly responsible for the biological activity; if one considers the MP2 results, only quind-3-t might be excluded because its relative energy in both solvents is too high (and the biological activity is exerted in a medium). It is not possible to predict a priori whether the presence of an IHB hampers or favours the exerting of biological activities by the given conformer (for instance, the antioxidant activity of polyphenols is enhanced by the presence of an IHB between two neighbour OHs [39,40]).

Besides the relative energies, this study has considered all the properties related to the molecular geometry (relevant torsion angles, parameters of the IHB) as well as other information related to the energetics (relative Gibbs free energies (sum of electronic and thermal free energies) in vacuo, free energy of solvation in chloroform and in water) and the harmonic vibrational frequencies of the O–H bond, in order to sufficiently provide comprehensive information about the conformers having an IHB and how their properties compare with those of the best conformer without IHB.

## 5. Conclusions

The current study reveals the presence of an intramolecular hydrogen bond in some conformers of quinine and quinidine. This result is confirmed at all the levels of theory utilized. The strength of this IHB is between weak and moderate.

## Figures and Tables

**Table 1:** Relevant dihedrale angles (°) of the most stable conformer of quinine and quinidine in previous conformational studies. The angles are denoted in the following way in the column headings: A for C11–C10–C9–C8, B for C10–C9–C8–N1, D for O20–C9–C8–N1 and E for H22–O20–C9–C8.

Reference	Quinine				Quinidine			
	A	B	D	E	A	B	D	E
[1]	67.6	−114.3		−65.4	−66.2	113.5		65.7
[15]	99.0	152.0	−83.0	174.0	−99.0	−153.0	83.0	−173.0

**Table 2:** Relative energy of the conformers of quinine and quinidine calculated within this work. Hartree-Fock (HF/6-31G(d,p)), Møller–Plesset Perturbation Theory (MP2/6-31+G(d,p) and Density Functional Theory (DFT/B3LYP/6-31+G(d,p)) results in vacuo and in chloroform and water solutions. The results in vacuo are from full optimization calculations, and the results in solution are from single point Polarisabel Continuum Model (PCM) calculations on the in vacuo-optimized geometries, at the same level of theory. The conformers are listed in order of increasing relative energies in the DFT results in vacuo.

Conformer	Relative Energy (kcal/mol)								
	HF			MP2			DFT		
	vac	chlrf	aq	vac	chlrf	aq	vac	chlrf	aq
quin-1-c	0.000	0.000	0.000	0.046	0.402	0.264	0.000	0.000	0.000
quin-1-t	2.024	1.569	2.641	2.175	2.061	1.405	1.249	0.995	0.809
quin-2-c	3.702	3.422	3.198	0.000	0.000	0.000	1.734	1.180	1.274
quin-2-t	5.192	4.797	4.712	1.648	1.594	1.434	2.265	1.988	2.266
quin-3-c	5.680	5.431	5.661	1.730	1.926	2.247	3.723	3.294	3.846
quin-3-t	7.837	7.234	6.760	4.337	4.164	3.506	4.599	4.698	4.816
quind-1-t	0.283	2.530	2.931	1.880	2.281	2.289	0.000	1.359	1.927
quind-2-t	0.206	3.342	2.759	0.030	3.355	2.119	0.755	2.863	2.275
quind-3-t	0.000	2.087	1.303	0.000	4.472	4.361	0.810	2.014	1.066
quind-1-c	2.410	0.720	1.856	2.641	0.000	0.824	1.696	0.000	0.989
quind-3-c	2.100	0.000	0.000	4.666	1.979	2.575	2.386	0.390	0.000
quind-2-c	3.331	0.715	1.376	3.684	0.089	0.000	3.125	0.900	1.156

**Table 3:** Relative Gibbs free energies (ΔG, sum of electronic and thermal free energies) of the calculated conformers of quinine and quinidine. HF/6-31G(d,p) and DFT/B3LYP/6-31+G(d,p) results in vacuo.

Conformer	Sum of Electronic and Thermal Free Energies (kcal/mol)		Conformer	Sum of Electronic and Thermal Free Energies (kcal/mol)	
	HF	DFT		HF	DFT
quin-1-c	0.000	0.000	quind-1-t	2.910	1.882
quin-1-t	5.907	3.666	quind-2-t	3.465	2.727
quin-2-c	4.827	2.937	quind-3-t	1.866	1.709
quin-2-t	1.539	1.787	quind-1-c	1.337	0.398
quin-3-c	6.669	4.895	quind-3-c	0.000	0.000
quin-3-t	8.271	6.305	quind-2-c	1.000	0.508

**Table 4:** Relevant dihedrale angles of the conformers of quinine and quinidine calculated within this work. HF/6-31G(d,p), MP2/6-31+G(d,p) and DFT/B3LYP/6-31+G(d,p) results in vacuo. The angles are denoted in the following way in the column headings: A for C11–C10–C9–C8, B for C10–C9–C8–N1, D for O20–C9–C8–N1 and E for H22–O20–C9–C8.

Conformer	HF				MP2				DFT			
	A	B	D	E	A	B	D	E	A	B	D	E
quin-1-c	101.6	152.1	−84.2	170.9	100.5	155.7	−81.3	169.0	99.3	153.6	−82.4	174.0
quin-1-t	101.2	154.8	−81.7	169.9	99.8	159.8	−77.7	167.5	99.6	155.8	−80.4	172.2
quin-2-c	99.1	−81.5	44.8	−30.0	100.4	−80.7	44.5	−29.4	99.8	−87.5	39.0	−24.1
quin-2-t	99.1	−81.5	44.8	−30.2	100.6	−81.9	43.2	−28.3	100.5	−88.8	37.7	−23.0
quin-3-c	−86.3	−81.6	49.0	−26.0	−89.0	81.1	48.9	−27.2	−81.6	−90.0	41.3	−23.0
quin-3-t	−87.5	−80.6	50.6	−29.0	−90.4	−79.3	50.6	−29.6	−83.4	−87.5	43.8	−25.7
quind-1-t	87.3	81.2	−49.9	27.1	86.7	83.2	−46.7	26.2	82.3	89.3	−41.9	24.3
quind-2-t	98.9	57.2	−71.1	−179.6	101.5	56.8	−70.6	174.0	100.1	56.3	−72.3	178.2
quind-3-t	82.5	−171.3	61.2	−83.0	91.2	159.4	33.6	−53.4	83.1	−169.0	63.8	−82.6
quind-1-c	88.8	80.2	−51.0	29.4	87.7	81.7	−48.2	28.3	84.8	86.9	−44.4	26.7
quind-3-c	84.2	−174.1	58.6	−79.3	91.2	159.4	33.6	−53.4	85.6	−174.7	58.2	−75.7
quind-2-c	98.6	56.2	−71.9	179.1	100.4	54.9	−72.2	173.2	100.4	53.3	−75.0	176.2

**Table 5:** Parameters of the intramolecular hydrogen bond (IHB) in the the calculated conformers of quinine and quinidine, which have an IHB. HF/6-31G(d,p), MP2/6-31+G(d,p) and DFT/B3LYP/6-31+G(d,p) results in vacuo. The distances (N···H and N···O) are in Å and the angle (NĤO) is in degrees.

Conformer	HF			MP2			DFT		
	N···H	N···O	NĤO	N···H	N···O	NĤO	N···H	N···O	NĤO
quin-2-c	2.112	2.726	121.0	1.989	2.677	125.2	1.980	2.675	125.8
quin-2-t	2.102	2.720	121.4	1.977	2.670	125.6	1.967	2.668	126.2
quin-3-c	2.187	2.785	120.0	2.082	2.748	123.6	2.000	2.700	125.9
quin-3-t	2.217	2.801	118.9	2.128	2.772	122.0	2.055	2.727	124.3
quind-1-t	2.194	2.788	119.8	2.039	2.721	124.9	2.010	2.703	125.8
quind-1-c	2.224	2.805	118.7	2.078	2.741	123.4	2.066	2.734	123.9
quind-3-t	2.856	2.979	88.2	2.280	2.740	107.9	2.903	3.044	89.0
quind-3-c	2.794	2.956	90.4	2.280	2.740	107.9	2.772	2.990	93.4

**Table 6:** Vibrational frequencies (harmonic approximation) of the O–H bond in the conformers of quinine and quinidine calculated within this work. HF/6-31G(d,p) and DFT/B3LYP/6-31+G(d,p) results in vacuo. The frequency values have been scaled by 0.9024 and 0.9857, respectively, and recommended for HF/6-31G(d,p) [25] and DFT/B3LYP/6 + 31(d,p) calculations in [26].

Quinine			Quinidine		
Conformer	Vibrational Frequencies (cm^−1^)		Conformer	Vibrational Frequencies (cm^−1^)	
	HF	DFT		HF	DFT
quin-1-c	4177	3766	quind-1-t	4114	3552
quin-1-t	4178	3767	quind-2-t	4177	3762
quin-2-c	4082	3514	quind-3-t	4195	3776
quin-2-t	4079	3507	quind-1-c	4106	3514
quin-3-c	4102	3513	quind-3-c	4197	3787
quin-3-t	4110	3545	quind-2-c	4174	3761

**Table 7:** Red shift of the O–H bonds in the calculated conformers of quinine and quinidine having an IHB. HF/6-31G(d,p) and DFT/B3LYP/6-31+G(d,p) results in vacuo.

Conformer	Red Shift of the Frequency of the OH Group (cm^−1^)	
	HF	DFT
quin-2-c	95	252
quin-2-t	99	259
quin-3-c	75	253
quin-3-t	68	221
quind-1-t	63	210
quind-1-c	68	248

**Table 8:** Dipole moment of the conformers of quinine and quinidine calculated within this work. HF/6-31G(d,p), MP2/6-31+G(d,p) and DFT/B3LYP/6-31+G(d,p) results in vacuo and in solution.

Conformer	Dipole Moment (Debye)								
	HF			MP2			DFT		
	vac	chlrf	aq	vac	chlrf	aq	vac	chlrf	aq
quin-1-c	2.789	3.336	3.999	3.007	3.759	4.679	2.903	3.610	4.423
quin-1-t	1.402	1.473	1.494	1.677	1.529	1.849	1.808	2.059	2.433
quin-2-c	4.459	5.106	5.857	4.707	5.440	6.329	4.733	5.648	6.623
quin-2-t	3.622	4.066	4.376	4.197	4.630	5.050	4.129	4.859	5.412
quin-3-c	4.465	5.113	5.876	4.518	5.473	6.380	4.405	5.258	6.175
quin-3-t	2.975	3.304	3.519	3.337	3.796	4.109	3.210	3.717	4.030
quind-1-t	3.059	3.457	3.882	3.266	3.816	4.417	3.319	3.934	4.542
quind-2-t	0.703	1.010	1.622	1.066	1.341	1.981	1.160	1.472	1.603
quind-3-t	1.987	2.340	2.772	2.837	3.359	4.104	2.325	2.823	3.299
quind-1-c	4.708	5.493	6.501	4.542	5.779	7.032	4.797	5.857	7.116
quind-3-c	4.242	4.979	5.893	4.515	5.161	6.228	4.235	5.771	7.049
quind-2-c	3.310	4.029	5.030	3.657	4.596	5.794	3.355	4.252	5.401

**Table 9:** Solvent effect (free energy of solvation, ΔG_solv_) for the calculated conformers of quinine and quinidine in chloroform (chlrf) and water (aq). Results from HF/6-31G(d,p), MP2/6-31+G(d,p) and DFT/B3LYP/6-31+G(d,p) single point PCM calculations on the in vacuo-optimized geometries.

Conformers	ΔG_solv_ (kcal/mol)					
	HF		MP2		DFT	
	chlrf	aq	chlrf	aq	chlrf	aq
quin-1-c	−0.95	−9.24	−1.28	−11.38	−0.74	−9.82
quin-1-t	−1.93	−10.94	−2.28	−13.11	−1.71	−11.14
quin-2-c	−0.89	−9.29	−1.87	−11.86	−0.92	−9.76
quin-2-t	−1.49	−9.83	−2.41	−12.63	−1.60	−10.35
quin-3-c	−0.68	−8.37	−1.53	−10.81	−0.71	−8.83
quin-3-t	−1.50	−10.07	−2.42	−13.02	−1.46	−10.19
quind-1-t	−0.79	−9.02	−1.49	−11.09	−0.76	−9.07
quind-2-t	−0.83	−10.04	−1.71	−12.85	−0.57	−10.06
quind-3-t	−0.03	−7.30	−0.64	−9.09	−0.03	−7.73
quind-3-c	−1.24	−9.88	−0.96	−9.70	−1.12	−10.40
quind-2-c	0.20	−7.48	−0.85	−10.47	0.35	−8.02
quind-1-c	−1.73	−11.45	−1.78	−11.60	−1.53	−11.67

**Table 10:** Electrostatic component (G_el_) of the free energy of solvation for the calculated conformers of quinine and quinidine in chloroform (chlrf) and water (aq). Results from HF/6-31G(d,p), MP2/6-31+G(d,p) and DFT/B3LYP/6-31+G(d,p) single point PCM calculations on the in vacuo-optimized geometries.

Conformers	G_el_ (kcal/mol)					
	HF		MP2		DFT	
	chlrf	aq	chlrf	aq	chlrf	aq
quin-1-c	−3.78	−15.42	−4.57	−18.15	−3.82	−16.43
quin-1-t	−4.23	−16.41	−5.04	−19.20	−4.31	−17.10
quin-2-c	−4.06	−15.93	−4.99	−18.48	−4.37	−16.89
quin-2-t	−4.18	−15.90	−2.41	−18.70	−4.52	−16.86
quin-3-c	−4.04	−15.45	−4.84	−17.91	−4.25	−16.30
quin-3-t	−4.38	−16.50	−5.24	−19.42	−4.59	−17.08
quind-3-t	−4.46	−17.53	−4.98	−18.63	−4.60	−18.30
quind-2-t	−4.44	−17.30	−1.71	−20.00	−4.49	−17.83
quind-1-t	−4.33	−16.21	−5.14	−18.62	−4.56	−16.75
quind-1-c	−4.02	−15.16	−4.74	−17.30	−4.23	−15.99
quind-2-c	−3.93	−15.56	−4.75	−18.36	−4.08	−16.58
quind-3-c	−4.45	−16.73	−4.65	−17.51	−4.65	−17.79
